# Integrating CT-based radiomics and deep learning for invasive prediction of ground-glass nodules in lung adenocarcinoma: a multicohort study

**DOI:** 10.1186/s13244-025-02156-6

**Published:** 2025-12-08

**Authors:** Hai Du, Jing Shen, Feng Chen, Kaifeng Wang, Lili Qin, Yijiang Hu, Yue Xiao, Xiulin Wang, Jianlin Wu

**Affiliations:** 1 Department of Radiology, Ordos Central Hospital, Ordos, 017000 Inner Mongolia China; 2 Department of Radiology, Affiliated Zhongshan Hospital of Dalian University, Dalian, 116001 Liaoning China; 3 Department of Oncology, Ordos Central Hospital, Ordos, 017000 Inner Mongolia China; 4 Fujian Medical University, Fuzhou, 350001 Fujian China; 5 Department of Radiology, Dalian Public Health Clinical Center, Dalian, 116031 Liaoning China; 6 Department of Radiology, Zhangjiagang Hospital affiliated to Soochow University / The First People’s Hospital of Zhangjiagang City, Zhangjiagang, 215600 Jiangsu China; 7 Clinical application department, Zhejiang Yizhun Intelligent Technology Co., Ltd, Lishui, 323010 Zhejiang China; 8 Stem Cell Clinical Research Center, The First Affiliated Hospital of Dalian Medical University, Dalian, 116021 Liaoning China; 9 Dalian Innovation Institute of Stem Cell and Precision Medicine, Dalian, 116085 Liaoning China

**Keywords:** Deep learning, Invasiveness, Lung adenocarcinoma, Multiple-instance learning, Radiomics

## Abstract

**Objectives:**

This study aimed to explore a multiple-instance learning (MIL) framework incorporating radiomics features and deep learning representations to predict the invasiveness of ground-glass nodules (GGNs) in lung adenocarcinoma (LUAD) using preoperative CT.

**Materials and methods:**

We retrospectively analyzed 1247 GGNs from 1182 LUAD patients across six hospitals, and divided them into training, validation and three test sets. According to postoperative pathological findings, the data were further classified into invasive and non-invasive subgroups. Five kinds of predictive models were developed: radiomics models, 3D deep learning models, 2.5D deep learning models, deep learning-based MIL (MIL-DL) models, and deep learning and radiomics-based MIL (MIL-DL-Rad) models. Model performance was evaluated using the area under the receiver operating characteristic curve (AUC), calibration curve, and decision curve analysis (DCA).

**Results:**

The MIL-DL-Rad model with the ExtraTrees classifier exhibited superior and consistent performance across all sets, achieving AUCs of 0.936, 0.881, 0.868, 0.926, and 0.918 in training, validation and external test sets. In contrast, the AUC performance of MIL-DL and radiomics models was relatively unstable. The calibration curve and DCA indicated that the integrated model achieved favorable predictive efficiency and clinical predictive benefits.

**Conclusions:**

The MIL-DL-Rad model showed better overall performance for invasiveness prediction of GGNs in LUAD patients, providing a novel perspective on feature fusion that can contribute to more accurate preoperative predictions in clinical practice.

**Critical relevance statement:**

Multi-instance learning integrating deep learning and radiomics enhances the prediction of ground-glass nodule (GGN) invasiveness and is expected to provide optimal preoperative clinical decision-making for lung adenocarcinoma patients.

**Key Points:**

- Ground-glass nodules invasiveness directly influences surgical strategies and prognosis.
- Multiple-instance learning framework integrates radiomics and deep learning features.
- Integrated model achieves superior accuracy and consistency in invasiveness prediction.

**Graphical Abstract:**

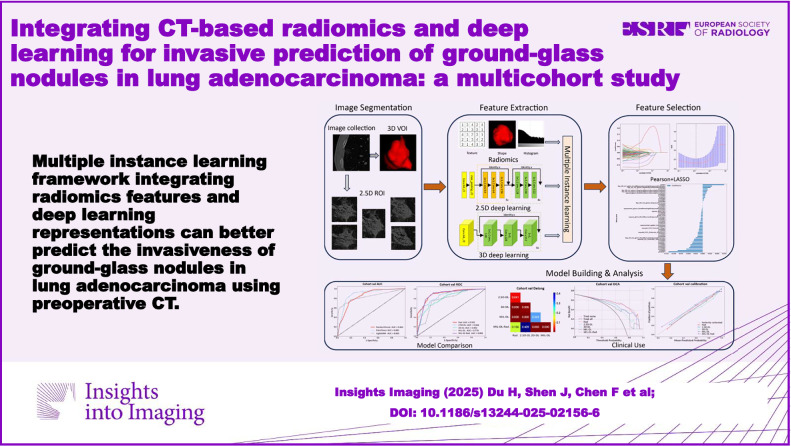

## Introduction

Lung cancer is one of the most prevalent cancers in terms of morbidity and mortality worldwide, among which lung adenocarcinoma (LUAD) is the most common histopathological subtype, and a significant proportion of LUAD cases present as ground-glass nodules (GGNs) on CT images [1, 2]. According to the 2021 World Health Organization guideline, LUAD is classified into precursor glandular lesions (PGL), minimally invasive adenocarcinoma (MIA), and invasive adenocarcinoma (IAC). PGL includes atypical adenomatous hyperplasia (AAH) and adenocarcinoma in situ (AIS), representing distinct stages in the progression of GGNs in LUAD [3, 4]. Previous studies have clearly demonstrated that different pathological subtypes of LUAD vary remarkably in treatment strategies and prognosis [5–8]. Non-invasive adenocarcinoma (NIAC), including AAH, AIS and MIA, typically requires conservative sublobar resection or long-term CT follow-up, with an almost 100% 5-year disease-free survival (DFS) rate. In contrast, IAC often necessitates more aggressive surgical treatment, such as lobectomy and extended lymph node dissection, and has a 5-year DFS rate of approximately 49–84% [8]. Therefore, accurate preoperative identification of the invasiveness of GGNs in LUAD is of great significance for guiding clinical decision-making and predicting patient prognosis.

Pathological examination of tissue sections is the gold standard for diagnosing the invasiveness of GGNs in clinical practice. However, it has notable limitations in precisely determining the tumor invasion area [9]. With the widespread application of high-resolution CT, preoperative CT evaluation of GGN invasiveness has become common. Although CT imaging features like nodule size, density, volume, maximum diameter, and CT value have been proposed as effective parameters for differentiating GGN invasiveness [9–13], these studies lack a unified feature standard. Most focus on single or combined imaging features, resulting in inconsistent conclusions and low inter-observer agreement.

The rapid development of artificial intelligence has led to the application of radiomics and deep learning in medical image analysis and diagnosis [14, 15]. Radiomics uses artificial intelligence techniques for high-throughput feature extraction, converting image data into quantitative features, which are then combined with nomograms, morphological features or other machine learning algorithms [16, 17]. Despite its potential in assessing GGN invasiveness, radiomic features are highly dependent on tumor segmentation, radiologists’ subjective judgment, radiation dose, and reconstruction algorithm, leading to poor reproducibility of the results [14, 18]. Deep learning, on the other hand, has shown superiority in image classification and target detection in an end-to-end manner without the need for manual feature extraction [17, 19, 20]. However, its black-box feature makes the decision-making process less interpretable. Nevertheless, deep learning features contain a large amount of original image information and can comprehensively capture the regional characteristics of the target dataset, providing a potential solution to the limitations of radiomics [20, 21].

Previous studies have explored the use of radiomics or deep learning in predicting GGN invasiveness [20, 22, 23], but most were single-center studies with limited sample sizes. Additionally, the integration of these two techniques in a multi-center setting, especially using multiple-instance learning (MIL), remains underexplored. In this multi-center study, we aim to fill this gap by developing and validating a MIL model that integrates radiomics and deep learning features. This model is based on preoperative CT data and is designed to evaluate its diagnostic efficacy in predicting the invasiveness of GGNs in LUAD, ultimately guiding personalized preoperative surgical planning. We also constructed traditional radiomics models, 2.5D and 3D deep learning models, and MIL models, integrating deep learning for comprehensive comparison.

## Materials and methods

### Study population

This retrospective study was approved by the Ethics Committee of Affiliated Zhongshan Hospital of Dalian University (No. 2021029, Dalian, China), and the requirement for written informed consent was waived. From January 2013 to June 2021, patients diagnosed with LUAD shown as GGNs and underwent lung resection at six hospitals, namely Affiliated Zhongshan Hospital of Dalian University, Affiliated Xinhua Hospital of Dalian University, the Second Hospital of Dalian Medical University, The Fifth People’s Hospital of Dalian, the Affiliated Hospital of Qingdao University and Zhangjiagang First People’s Hospital, were initially considered for eligibility assessment. A total of 1415 GGNs were diagnosed and pathologically confirmed with IAC, MIA, AIS, or AAH.

The inclusion criteria were as follows: (1) LUAD patients with GGNs visible on CT images who underwent lung resection; (2) GGNS with a maximum diameter < 3 cm; (3) patients with preoperative thin-section CT images of 1–1.25 mm slice thickness; (4) patients confirmed as having primary LUAD. The exclusion criteria were as follows: (1) a history of radiotherapy, chemotherapy, or biopsy prior to the baseline CT; (2) an interval between preoperative CT examination and surgery exceeding 2 weeks; (3) severe motion artifacts in the CT images; (4) incomplete preoperative chest CT, clinical, or pathological data.

**Fig. 1:**
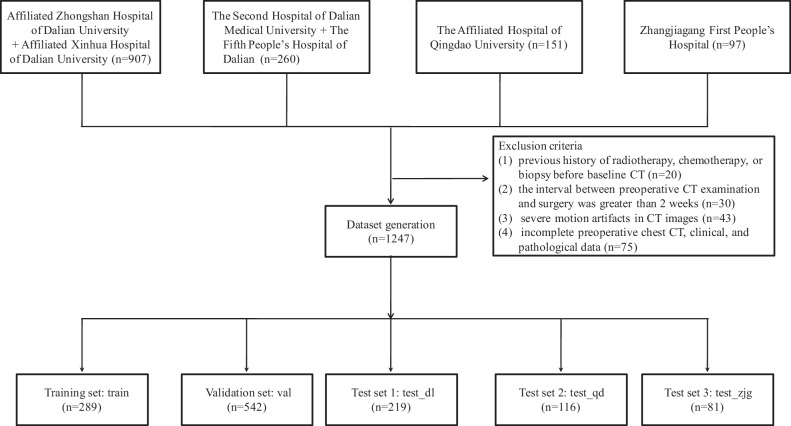
The enrollment flowchart of this study

In the end, 1247 GGNs from 1182 patients were included. The data from the first two hospitals were divided into the training set and the validation set. The data from two other Dalian hospitals constituted the Dalian area test set (test_dl). The remaining two hospitals provided data for two external test sets (test_qd and test_zjg). Each set was further divided into invasive and non-invasive groups. The training set was used to construct the prediction models, while the other sets were employed to verify the predictive efficiency. The enrollment flowchart of this study is shown in Fig. 1.

### CT image evaluation

The chest CT images were acquired in the Digital Imaging and Communications in Medicine (DICOM) format. Subsequent image analysis and measurements were conducted with the RadiAnt DICOM Viewer (version 2023.1). The CT images were independently reviewed by two radiologists, who had 11 and 13 years of experience in chest imaging diagnosis, and were blinded to the pathological results. Each radiologist measured each GGN twice, and the average value was calculated. Subsequently, the average of the two radiologists’ measurements was taken as the final result. In cases of discrepancies, the differences were resolved by consulting the superior chief physician. Further details regarding the image acquisition parameters are provided in the supplementary material.

### Nodule segmentation

For the region of interest (ROI) definition, the two radiologists manually delineated lung lesions using ITK-SNAP software (version 4.0.0) slice by slice, taking care to avoid adjacent blood vessels, bronchi, and mediastinal or chest wall structures. Then, the ROI of each slice was fused into a volume of interest (VOI). To ensure intra- and inter-observer consistency, an intra-/inter-class correlation coefficient (ICC) assessment was required. A third senior radiologist resolved any disagreements. When the ICC value is greater than 0.85, the reproducibility is considered satisfactory.

### Model development

#### Radiomic model

1834 radiomic features were extracted using the Pyradiomics toolbox, including 360 first-order features, 14 shape features, and 1460 texture features. Feature screening was carried out by *t*-test (*p* < 0.05), Pearson correlation (> 0.9), and the least absolute shrinkage and selection operator (LASSO) regression. The screened features were employed to construct prediction models using machine learning algorithms, including random forest (RF), extremely randomized trees (ExtraTrees), and light gradient boosting machine (LightGBM).

#### 2.5D deep learning model

The maximum VOI section of the original 3D image was designated as the benchmark. 2D image slices adjacent to the benchmark section (at distances of *±* 1, *±* 2, and *±* 4 from the benchmark) were selected to form 2.5D data. Slice-level performance will eventually be incorporated into the MIL model. To improve the generalization capabilities of the models, data augmentation techniques such as random cropping, random brightness adjustment, random rotation, horizontal flipping, and vertical flipping were applied.

In this part, five 2.5D deep learning (2.5D-DL) models, namely DenseNet121, DenseNet201, ResNet50, ResNet101, and VGG19 were employed. By leveraging the transfer learning strategy, the model parameters were initialized by loading the weight parameters of the model pre-trained on ImageNet. Only the classification network for the target task was added, avoiding the need to train the model from scratch.

#### 3D deep learning model

In this part, three 3D deep learning (3D-DL) models, including ResNet50-3D, DenseNet121-3D and ShuffleNet-3D, were adopted to extract features. Similar to 2.5D-DL models, an augmentation strategy was applied, and transfer learning was utilized to alleviate the over-fitting problem and speed up the training process. The parameters of the pre-trained MedicalNet model were used for the model initialization.

#### Multiple-instance learning model

Inspired by the fusion methods in pathomics, we employed MIL-based methods of Predict Likelihood Histogram (PLH) and Bag of Words (BoW) pipeline to fuse the features of different deep learning models and radiomics [24, 25]. Specifically, the features extracted by 2.5D-DL and 3D-DL models will be fused to build the deep learning-based MIL (MIL-DL) model. Then, the multi-instance features fused by deep learning were further combined with the features of radiomics to construct the deep learning and radiomics-based MIL (MIL-DL-Rad) model.

### Model evaluation

**Fig. 2:**
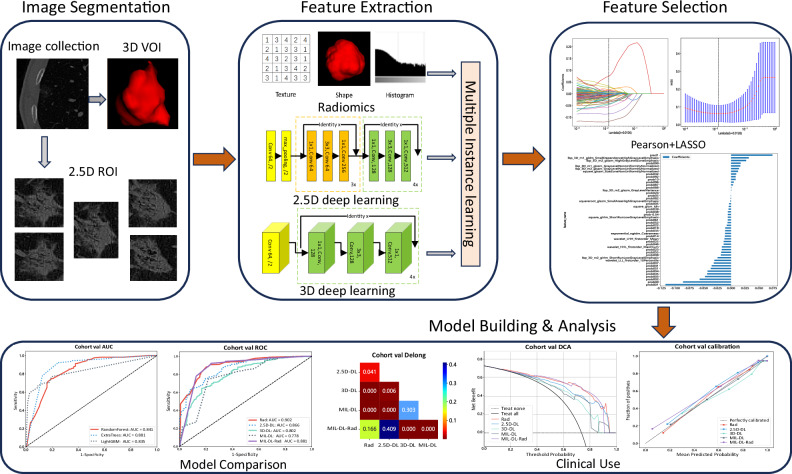
Flowchart of feature extraction, model establishment and performance evaluation

Variables were compared using the Mann–Whitney *U*-test, Student’s *t*-test, or Chi-square test, as appropriate. Normally distributed data were presented by the mean ± standard deviation. A *p-*value < 0.05 was considered statistically significant. Model predictive performance was evaluated using the receiver operating characteristic (ROC) curve, area under the ROC curve (AUC), accuracy, sensitivity, specificity, positive predictive value (PPV), and negative predictive value (NPV). The Delong test, net reclassification improvement (NRI), and integrated discrimination improvement (IDI) were employed to compare model performance. Decision curve analysis (DCA) and calibration curve were used to assess the accuracy and practical value of the models. Further details, including these metrics, computer configuration, programming environment and model optimization, are provided in the supplementary material. A flowchart of our study, including feature extraction, model establishment and performance evaluation, is shown in Fig. 2.

## Results

### Patient demographics and clinical characteristics

This study included 1247 GGN lesions from LUAD patients. The age of the patients ranged from 21 to 83 years (56.50 *±* 10.80). Based on pathological results, the GGNs were divided into an invasive group (*n* = 841, 67.44%, Male = 302, Female = 539, Age = 60.71 *±* 8.94) and a non-invasive group (*n* = 406, 32.56%, Male = 109, Female = 297, Age = 52.28 *±* 12.67). The invasive and non-invasive groups exhibited significant differences (*p* *<* 0.01) in age, volume, maximum diameter, minimum diameter, average diameter, and CT value. There was no significant difference in the location of GGNs (*p* *>* 0.05). GGNs most commonly occurred in the right upper lobe with 421 cases (33.76%), of which 300 cases (35.67%) were in the invasive group and 121 cases (29.8%) in the non-invasive group. Followed by the left upper lobe with 353 cases (28.31%), including 242 cases (28.78%) and 111 cases (27.34%) in the two groups. please refer to Table 1 and Table 2 for details.

**Table 1:** Demographic and semantic CT features of patients in the train and validation cohorts

Characteristics	Training cohort (*n* = 289)		*p-*value	Validation cohort (*n* = 542)		*p-*value
	NInv Group (*n* = 93)	Inv Group (*n* = 196)		NInv Group (*n* = 143)	Inv Group (*n* = 399)	
Age	52.15 ± 13.45	60.37 ± 8.21	< 0.01	55.26 ± 11.98	61.04 ± 8.98	< 0.01
Gender						
Female	71 (76.34)	142 (72.45)	0.57	108 (75.52)	235 (58.90)	< 0.01
Male	22 (23.66)	54 (27.55)		35 (24.48)	164 (41.10)	
GGN						
Nodule type						
mGGN	37 (39.78)	172 (87.76)	< 0.001	55 (38.46)	330 (82.71)	< 0.01
pGGN	56 (60.22)	24 (12.24)		88 (61.54)	69 (17.29)	
Volume	339.53 ± 338.61	2261.74 ± 3039.38	< 0.01	352.08 ± 264.87	2050.73 ± 2617.14	< 0.01
Maximum diameter	10.68 ± 3.84	19.09 ± 8.07	< 0.01	10.14 ± 2.96	18.45 ± 7.50	< 0.01
Minimum diameter	7.97 ± 2.91	13.37 ± 5.56	< 0.01	7.55 ± 2.32	13.33 ± 5.45	< 0.01
Average diameter	9.35 ± 3.23	16.27 ± 6.44	< 0.01	8.85 ± 2.53	15.91 ± 6.18	< 0.01
Left lower lobe	16 (17.20)	32 (16.33)	0.36	28 (19.58)	58 (14.54)	0.09
Left upper lobe	30 (32.26)	62 (31.63)		37 (25.87)	108 (27.07)	
Right lower lobe	17 (18.28)	26 (13.27)		19 (13.29)	60 (15.04)	
Right middle lobe	9 (9.68)	12 (6.12)		18 (12.59)	27 (6.77)	
Right upper lobe	21 (22.58)	64 (32.65)		41 (28.67)	146 (36.59)	

*NInv* non-invasive, *Inv* invasive

**Table 2:** Demographic and semantic CT features of patients in the testing cohorts

Characteristics	Test dl cohort (*n* = 219)		*p-*value	test qd cohot (*n* = 116)		*p-*value	test zjg cohort (*n* = 81)		*p-*value
	NInv Group (*n* = 77)	Inv Group (*n* = 142)		Ninv Group (*n* = 50)	Inv Group (*n* = 66)		Ninv Group (*n* = 43)	Inv Group (*n* = 38)	
Age	52.57 ± 11.86	61.15 ± 8.75	< 0.01	50.32 ± 10.95	57.68 ± 11.04	< 0.01	44.40 ± 13.17	62.66 ± 7.85	< 0.01
Gender									
Female	52 (67.53)	90 (63.38)	0.64	33 (66.00)	50 (75.76)	0.344	33 (76.74)	22 (57.89)	0.16
Male	25 (32.47)	52 (36.62)		17 (34.00)	16 (24.24)		10 (23.26)	16(42.11)	
GGN									
Nodule type									
mGGN	26 (33.77)	97 (68.31)	< 0.01	16 (32.00)	41 (62.12)	0.002	13 (30.23)	32 (84.21)	< 0.01
pGGN	51 (66.23)	45 (31.69)		34 (68.00)	25 (37.88)		30 (69.77)	6 (15.79)	
Volume	388.72 ± 216.31	2345.89 ± 2986.48	< 0.01	407.46 ± 337.58	740.57 ± 784.86	0.04	222.51 ± 123.99	1788.62 ± 2890.79	< 0.01
Maximum diameter	9.61 ± 2.90	18.96 ± 7.40	< 0.01	9.13 ± 2.89	13.27 ± 4.90	< 0.01	8.94 ± 1.71	18.96 ± 8.41	< 0.01
Minimum diameter	7.57 ± 2.49	14.08 ± 5.35	< 0.01	7.25 ± 2.17	9.72 ± 3.67	< 0.01	7.18 ± 1.43	12.63 ± 5.02	< 0.01
Average diameter	8.57 ± 2.65	16.58 ± 6.16	< 0.01	8.24 ± 2.44	11.52 ± 4.09	< 0.01	8.14 ± 1.47	15.84 ± 6.48	< 0.01
CT value	−482.86 ± 174.43	−478.34 ± 175.81	0.74	−597.15 ± 95.85	−520.98 ± 132.01	0.02	−549.35 ± 103.16	−379.64 ± 221.90	< 0.01
Location									
Left lower lobe	12 (15.58)	18 (12.68)	0.33	5 (10.00)	8 (12.12)	0.59	10 (23.26)	4 (10.53)	0.37
Left upper lobe	16 (20.78)	49 (34.51)		16 (32.00)	14 (21.21)		12 (27.91)	9 (23.68)	
Right lower lobe	14 (18.18)	23 (16.20)		9 (18.00)	10 (15.15)		6 (13.95)	4 (10.53)	
Right middle lobe	6 (7.79)	10 (7.04)		3 (6.00)	3 (4.55)		2 (4.65)	4 (10.53)	
Right upper lobe	29 (37.66)	42 (29.58)		17 (34.00)	31 (46.97)		13 (30.23)	17 (44.74)	

*NInv* non-invasive, *Inv* invasive

### MIL-DL-Rad feature selection

**Fig. 3:**
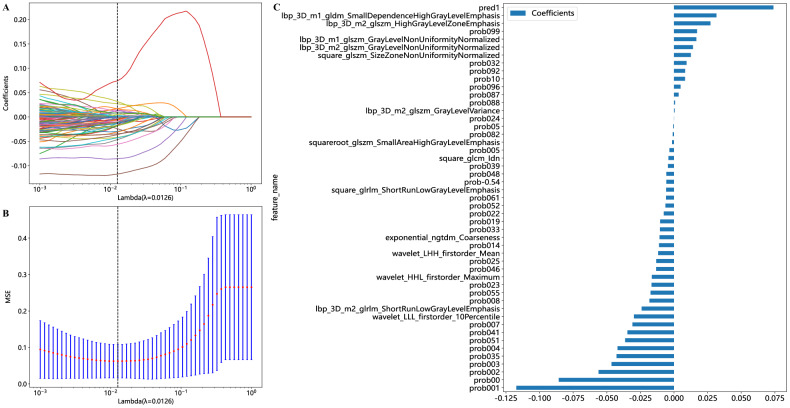
Feature screening in deep learning and radiomic-based multiple-instance learning (MIL-DL-Rad) models using the least absolute shrinkage and selection operator (LASSO). **A** Coefficient plot during LASSO cross-validation. **B** MSE cross-validation plot. **C** Feature plot of the 49 non-zero coefficients screened out

In the MIL-DL-Rad model, we obtained the multi-instance features fused by models of 2.5D-DL, 3D-DL and radiomics. Through *t*-test, Pearson correlation and LASSO regression, we finally screened out 49 multi-instance features for modeling. Figure 3 gives an illustration of feature screening using LASSO regression, compressing the coefficients of unimportant features to zero through regularization, retaining only the most critical features for model prediction (*λ* = 0.0126). The details of feature selection can be found in the supplementary file.

### MIL-DL-Rad model performance

**Fig. 4:**
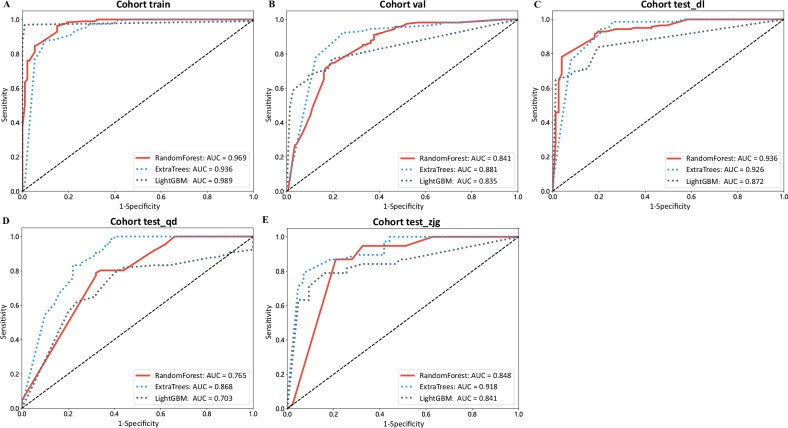
Comparison of receiver operating characteristic (ROC) curves in deep learning and radiomic-based multiple-instance learning (MIL-DL-Rad) models. Panels **A**–**E** show ROC curve comparison in the train, val, test_dl, test_qd, and test_zjg cohorts

We compared the performance of MIL-DL-Rad models in Fig. 4 and Table 3. In the training set, the LightGBM model achieved the best performance with an AUC of 0.989. In the validation set, the ExtraTrees model had the highest AUC of 0.881, followed by the RandomForest model with an AUC of 0.841. In test sets, the ExtraTrees model performed the best on test_qd and test_zjg with AUCs of 0.868 and 0.918, respectively. In the test_dl set, the RandomForest model had the highest AUC of 0.936, followed by the ExtraTrees model with an AUC of 0.926. Taking into account other metrics, such as accuracy, sensitivity, and specificity, we concluded that the ExtraTrees model has the optimal performance for further comparison.

**Table 3:** Performance comparison of deep learning and radiomic-based multiple-instance learning (MIL-DL-Rad) models using machine learning classifiers

Model name	Accuracy	AUC	95% CI	Sensitivity	Specificity	PPV	NPV	Sets
RandomForest	0.927	0.969	0.951–0.988	0.964	0.849	0.931	0.919	train
RandomForest	0.762	0.841	0.800–0.882	0.747	0.810	0.914	0.532	val
RandomForest	0.845	0.936	0.902–0.970	0.782	0.974	0.974	0.705	test dl
RandomForest	0.741	0.765	0.683–0.846	0.788	0.680	0.765	0.708	test qd
RandomForest	0.827	0.848	0.766–0.930	0.868	0.810	0.786	0.872	test zjg
ExtraTrees	0.886	0.936	0.902–0.970	0.878	0.913	0.950	0.778	train
ExtraTrees	0.880	0.881	0.847–0.916	0.922	0.768	0.915	0.779	val
ExtraTrees	0.890	0.926	0.888–0.965	0.944	0.792	0.893	0.884	test dl
ExtraTrees	0.810	0.868	0.799–0.936	0.833	0.780	0.833	0.780	test qd
ExtraTrees	0.864	0.918	0.859–0.976	0.789	0.930	0.909	0.833	test zjg
LightGBM	0.983	0.989	0.979–0.999	0.980	0.989	0.995	0.958	train
LightGBM	0.736	0.835	0.805–0.865	0.677	0.915	0.951	0.500	val
LightGBM	0.826	0.872	0.829–0.915	0.838	0.816	0.888	0.729	test dl
LightGBM	0.707	0.703	0.607–0.799	0.788	0.750	0.722	0.682	test qd
LightGBM	0.815	0.841	0.750–0.932	0.789	0.878	0.811	0.818	test zjg

*AUC* area under the receiver operating characteristic curve, *PPV* positive predictive value, *NPV* negative predictive value, *CI* confidence interval

### Performance comparison

To better compare the performance of various models in predicting GGN invasiveness in LUAD patients, we only selected the best-performing model (referring to the principle of test set) as the final model, and the performance comparison is shown in Table 4. The specific selection criteria are defined as follows: for the radiomics, the ExtraTrees-based model is selected (termed as Rad); for the 2.5D deep learning, the VGG19 model is selected; for the 3D deep learning, the ShuffleNet model is chosen; for the MIL-DL model, the ExtraTrees-based model is selected; and for the MIL-DL-Rad model, the ExtraTrees-based model is selected. It should be noted that for fairness, the 2.5D model only reports the performance of its benchmark section for patient-level comparison with other models, while the MIL-DL model involves the slice-level performance of all image slices from 2.5D models. Due to the space limitation, the performance comparison within radiomics, 2.5D-DL, 3D-DL, and MIL-DL models can be found in the supplementary material.

**Table 4:** Performance comparison of radiomics (Rad), 2.5D deep learning (2.5D-DL), 3D deep learning (3D-DL), deep learning-based multiple-instance learning (MIL-DL) and deep learning and radiomic-based multiple-instance learning (MIL-DL-Rad) models

Model name	Accuracy	AUC	95% CI	Sensitivity	Specificity	PPV	NPV	Sets
Rad	0.889	0.935	0.9032–0.9661	0.893	0.891	0.941	0.796	train
2.5D-DL	0.7234	0.836	0.7896–0.8819	0.628	0.925	0.946	0.541	train
3D-DL	0.986	0.992	0.9792–1.0000	0.990	0.978	0.990	0.978	train
MIL-DL	0.941	0.982	0.9711–0.9927	0.934	0.957	0.979	0.873	train
MIL-DL-Rad	0.886	0.936	0.9020–0.9700	0.878	0.913	0.950	0.778	train
Rad	0.860	0.902	0.8735–0.9301	0.882	0.803	0.924	0.708	val
2.5D-DL	0.768	0.866	0.8338–0.8985	0.754	0.804	0.915	0.540	val
3D-DL	0.795	0.802	0.7597–0.8441	0.880	0.559	0.848	0.625	val
MIL-DL	0.756	0.778	0.7322–0.8242	0.747	0.800	0.906	0.526	val
MIL-DL-Rad	0.880	0.881	0.8465–0.9163	0.922	0.768	0.915	0.779	val
Rad	0.886	0.936	0.9035–0.9686	0.887	0.883	0.933	0.810	test dl
2.5D-DL	0.849	0.930	0.8981–0.9624	0.838	0.870	0.922	0.744	test dl
3D-DL	0.822	0.873	0.8199–0.9252	0.937	0.610	0.816	0.839	test dl
MIL-DL	0.822	0.874	0.8255–0.9227	0.810	0.855	0.906	0.707	test dl
MIL-DL-Rad	0.890	0.926	0.8877–0.9650	0.944	0.792	0.893	0.884	test dl
Rad	0.784	0.828	0.7551–0.9006	0.924	0.600	0.753	0.857	test qd
2.5D-DL	0.845	0.866	0.7964–0.9348	0.955	0.700	0.808	0.921	test qd
3D-DL	0.690	0.762	0.6746–0.8490	0.667	0.720	0.759	0.621	test qd
MIL-DL	0.741	0.796	0.7155–0.8757	0.712	1.000	0.810	0.672	test qd
MIL-DL-Rad	0.810	0.868	0.7987–0.9364	0.833	0.780	0.833	0.780	test qd
Rad	0.889	0.914	0.8452–0.9822	0.842	0.952	0.914	0.870	test zjg
2.5D-DL	0.889	0.894	0.8130–0.9746	0.789	0.977	0.968	0.840	test zjg
3D-DL	0.852	0.942	0.8828–1.0000	0.711	0.977	0.964	0.792	test zjg
MIL-DL	0.877	0.882	0.8092–0.9558	0.921	1.000	0.833	0.923	test zjg
MIL-DL-Rad	0.864	0.918	0.8591–0.9763	0.789	0.930	0.909	0.833	test zjg

*AUC* area under the receiver operating characteristic curve, *PPV* positive predictive value, *NPV* negative predictive value, *CI* confidence interval

In the training, validation, and three test sets, the AUCs of MIL-DL-Rad models ranked third, second, first, second, and first, respectively. Even when ranked third, it achieved a fairly high value of 0.936, demonstrating relatively stable performance. AUC values quantify the model’s ability to distinguish invasive from non-invasive GGNs. MIL-DL-Rad with a higher AUC indicates better discrimination of tumor invasiveness, which is crucial for clinical decision-making. In contrast, the performance of 2.5D-DL and 3D-DL models ranked lower, especially on the test set. Radiomics showed comparable performance across all sets, with high accuracy, AUC, and balanced sensitivity and specificity. Similar to 2.5D-DL and 3D-DL models, the MIL-DL model only performed well on the training set.

**Fig. 5:**
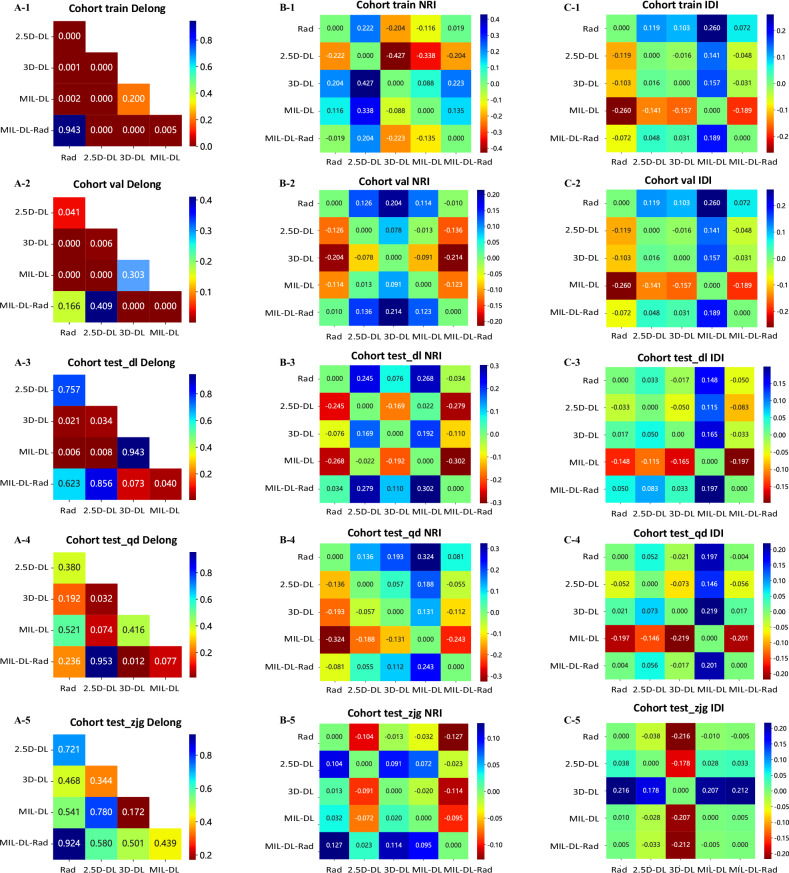
Comparison of Delong test, net reclassification improvement (NRI) and integrated discrimination improvement (IDI) between radiomics (Rad), 2.5D deep learning (2.5D-DL), 3D deep learning (3D-DL), deep learning-based multiple-instance learning (MIL-DL), and deep learning and radiomic-based multiple-instance learning (MIL-DL Rad) models. Panels **A**-1 to **A**-5: Delong test results; **B**-1 to **B**-5: NRI values; **C**-1 to **C**-5: IDI values

In the training set, Delong test (Fig. 5) shows MIL-DL-Rad has statistically significant differences from 2.5D-DL, 3D-DL, and MIL-DL models (*p* *<* 0.05); in the validation set, MIL-DL-Rad is significantly different from 3D-DL and MIL-DL (*p* *<* 0.05); the difference between MIL-DL-Rad and 3D-DL models in the test_qd set is statistically significant (*p* *<* 0.05); the difference between MIL-DL-Rad and MIL-DL models in the test_dl set is statistically significant (*p* *<* 0.05); in the test_zjg set, the differences between MIL-DL-Rad and Rad, 2.5D-DL, 3D-DL, and MIL-DL models are not statistically significant (*p* *>* 0.05).

**Fig. 6:**
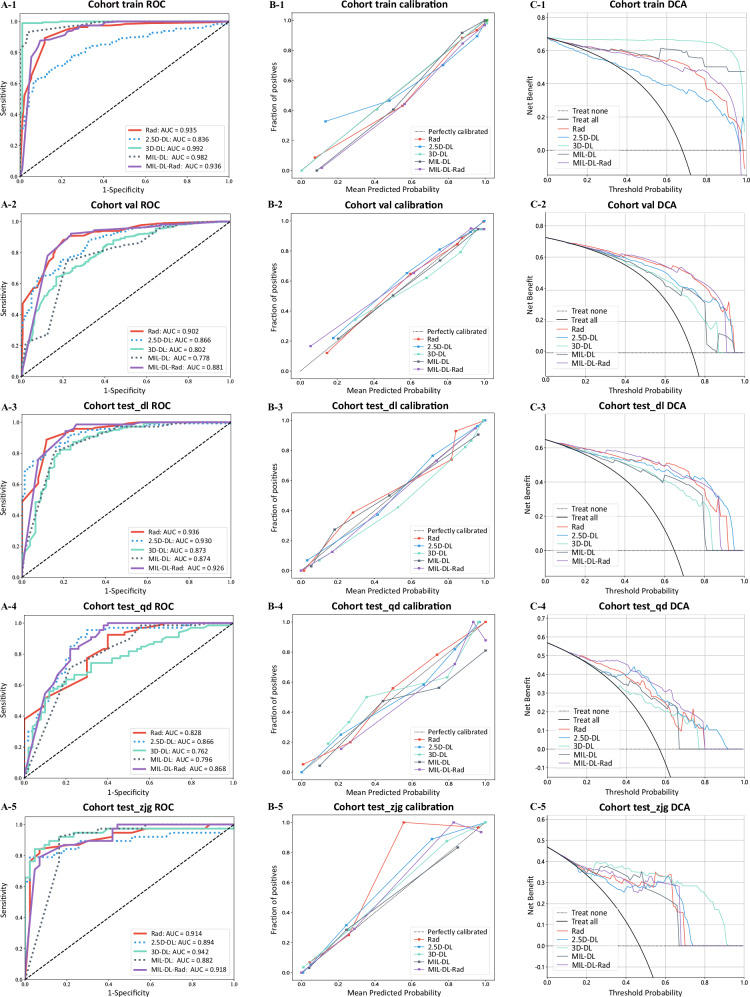
Comparison of receiver operating characteristic (ROC) curves, calibration curves, and decision curve analysis (DCA) in radiomics (Rad), 2.5D deep learning (2.5D-DL), 3D deep learning (3D-DL), deep learning-based multiple-instance learning (MIL-DL), and deep learning and radiomic-based multiple-instance learning (MIL-DL Rad) models. Panels **A**-1 to **A**-5: ROC curves; **B**-1 to **B**-5: calibration curves; **C**-1 to **C**-5: DCA curves.

Most of the NRI and IDI values are significantly positive, indicating that the MIL-DL-Rad model improves the prediction performance compared with other models (Fig. 5). The calibration curves in Fig. 6A indicate that the predicted probability of the MIL-DL-Rad model agrees well with the actual probability, reflecting accurate model calibration that supports individualized clinical decision-making. The DCA in Fig. 6B shows that in most threshold probability ranges, the MIL-DL-Rad model achieved higher clinical net benefit in validation and test sets, highlighting its practical utility for guiding treatment interventions.

## Discussion

This study developed five models based on radiomics, deep learning, and multiple-instance learning, aiming to explore their diagnostic efficacy for GGN invasiveness in LUAD using preoperative CT. The MIL-DL-Rad model, integrating deep learning and radiomics, consistently achieved stable performance and showed greater stability compared to other models, which performed inconsistently or well only on specific datasets. Moreover, it demonstrated statistically significant superiority over other models in most comparisons via DeLong tests (*p* < 0.05), and showed improved clinical utility via NRI/IDI and DCA.

Accurate preoperative prediction of invasiveness is essential for optimizing surgical strategies and improving outcomes. Most GGNs are slow-growing and indolent, with MIAs showing a ten-year recurrence-free rate [26]. Non-invasiveness was defined by the tumor’s solid component, guiding sublobar resection [27]. However, postoperative pathology often reveals IAC misdiagnosed as non-invasive, highlighting the importance of accurate preoperative prediction. It also facilitates rational follow-up, e.g., AIS volume doubling time was 567 days, AAH was 988, and pure GGN was 1448 [28, 29], suggesting annual follow-up for non-invasive GGNs. Accurate prediction aids in selecting less invasive surgeries (wedge, segment, subsegment resection), thereby preserving lung function and minimizing patient trauma [30]. Early IAC diagnosis can also improve survival rates, as studies have shown that the progression-free survival rate for IAC patients is 74.6% [31]. Prediction also assists intraoperative frozen section, which struggles with small GGNs, while CT outperforms intraoperative frozen techniques in predicting PIL and IAC in early-stage LUAD [32]. In particular, the combined model established by intraoperative freezing and radiomics performed better in diagnosis and could provide support for intraoperative decision-making [33].

Radiomics has emerged as a powerful tool since 2012, revealing disease characteristics by extracting quantitative features from medical images [34]. These features, though not easily observable by the naked eye, reflect tumor heterogeneity. Invasive GGNs exhibit greater heterogeneity than non-invasive ones, making radiomics a valuable tool for identifying GGN invasiveness. In this study, high-throughput radiomics features were extracted, with high-order features dominating. This finding aligns with [35], high-order features may better capture the heterogeneous changes of GGNs. We found that the ExtraTrees-based radiomics model performed particularly well. Similar results were reported by [36], who used gender and radiomics features to distinguish AIS/MIA from IAC, achieving AUC values of 0.96 and 0.90 in the training and validation sets. Zhang et al used radiomics models to predict the invasiveness of sub-centimeter GGNs, with the XGBoost model achieving an AUC of 0.874 [37]. Wang et al found that multimodal radiomics using dual-energy CT had good predictive ability, with a combined model’s AUC of 0.887 [38]. Compared to single-center radiomics studies, this study’s use of data from multiple hospitals (covering various CT machines and reconstruction algorithms) increased model robustness and prediction efficiency.

3D deep learning networks were utilized in this study. Park et al achieved AUC values of 0.914 and 0.833 in training and validation sets using a 3D deep learning model for GGN invasiveness identification [30]. Although the ShuffleNet-based model in this study achieved an AUC of only 0.783 in the validation set, it performed very well on other datasets. The 2.5D model combines 2D and 3D advantages, providing more context and spatial information. Kim et al designed 2.5D and 3D deep learning models, with the 2.5D model achieving an AUC of 0.921 [39]. However, this study was single-center and only used DenseNet121. This study expanded on this approach by incorporating multiple models and datasets. Standard algorithms for image thin-layer reconstruction may also affect accuracy. The test_qd set, using multiple manufacturers’ machines, showed poor performance with 3D networks, likely due to difficulties in effectively using the generated 3D data. These results highlight the importance of using multiple centers, manufacturers, and reconstruction algorithms to build robust models.

MIL is a weakly supervised learning method suitable for tasks with incomplete or inaccurate training data labels [40]. MIL models focus on identifying critical diagnostic areas while avoiding irrelevant ones, saving computing resources and improving efficiency. It can be combined with traditional machine learning algorithms to solve specific tasks. For example, MIL-based support vector machines can identify high-risk subregions in glioblastoma associated with patient survival [41], and MIL-based attention models can preoperatively distinguish luminal and non-luminal breast cancers at early stages using ultrasound and whole slide images [25]. In this study, both MIL-DL and MIL-DL-Rad achieved better performance in the ExtraTrees model. MIL-DL-Rad has the highest AUC (0.868) in the test_qd, while radiomics has the highest AUC (0.936) in the test_dl, followed by MIL-DL Rad with an AUC of 0.926. These results align with previous studies [42, 43], confirming that fusing radiomics and deep learning features can improve prediction performance. This study also demonstrated the potential of MIL-based methods in predicting GGN invasiveness. By aggregating PLH and BoW channels, the overall image features can be more comprehensively represented [24].

Moreover, inspired by this study on quantitative and semantic feature-based modeling [44], we developed a separate clinical model using analogous clinical features (e.g., age, nodule type). The performance of the RF-based clinical model on the training, test_dl, and test_zjg cohorts was comparable to that of the quantitative-semantic model [44], but it remained consistently lower than that of both radiomics and MIL-DL-Rad models. Furthermore, we also explored the performance of the joint model by integrating the clinical and MIL-DL-Rad modes in a post-fusion manner using a logistic regression model, demonstrating that clinical information can effectively enhance the robustness and discriminative power of the developed model. Please refer to the supplementary material for more details.

Despite achieving some anticipated results, this study still has several limitations. One of the limitations is about the inter-set imbalance of the datasets: the relatively small training set may impact model optimization, while the varying sizes of the external test sets (particularly those with limited samples) restrict the statistical power of the evaluation results and the robustness of generalizability interpretations, future work will pursue more balanced external cohorts through prospective data collection or multi-center collaborations. As a retrospective analysis, it may be subject to selection bias. Future research should include prospective randomized controlled studies to verify the role of the MIL framework in improving prediction accuracy. This study used manual delineation to segment the lesion, and next will explore the automatic segmentation to improve efficiency and reduce human differences. This study focused on preoperative CT data for predicting GGN invasiveness. Future research could incorporate pathomics and other clinical data to construct a more comprehensive model, potentially enhancing the effectiveness and applicability of GGN invasiveness prediction and improving patient outcomes in LUAD diagnosis and treatment.

## Conclusion

This multi-center study demonstrates that the proposed MIL-DL-Rad framework, integrating deep learning and radiomics within a multiple-instance learning paradigm, achieves superior and remarkably robust performance across multiple cohorts for predicting GGN invasiveness in LUAD patients. The proposed MIL-DL-Rad framework significantly outperforms and overcomes the instability limitations of models based on single-modality features or basic deep learning fusion approaches. This breakthrough validates the power of complementary feature fusion under weak supervision, providing clinicians with a highly reliable tool for preoperative risk stratification. This study provides a new perspective on feature fusion in the field of GGN invasiveness prediction, which may assist clinicians in making more accurate preoperative predictions and personalized surgical plans.

## Supplementary information

ELECTRONIC SUPPLEMENTARY MATERIAL

## Data Availability

All raw data and materials related to this study can be obtained by contacting the corresponding author.

## Abbreviations

AAH: Atypical adenomatous hyperplasia
AIS: Adenocarcinoma in situ
AUC: Area under the receiver operating characteristic curve
BoW: Bag of words
DCA: Decision curve analysis
DFS: Disease-free survival
DL: Deep learning
ExtraTrees: Extremely randomized trees
GGN: Ground-glass nodule
IAC: Invasive adenocarcinoma
ICC: Intra/inter-class correlation coefficient
IDI: Integrated discrimination improvement
LASSO: Least absolute shrinkage and selection operator
LightGBM: Light gradient boosting machine
LUAD: Lung adenocarcinoma
MIA: Minimally invasive adenocarcinoma
MIL: Multiple-instance learning
NPV: Negative predictive value
NRI: Net reclassification improvement
PGL: Precursor glandular lesions
PLH: Predict likelihood histogram
PPV: Positive predictive value
RF: Random forest
ROC: Receiver operating characteristic
ROI: Region of interest
VOI: Volume of interest
