# Supplementary material for: Integrating CT-based radiomics and deep learning for invasive prediction of ground-glass nodules in lung adenocarcinoma: a multicohort study
Source: Insights Imaging. 2025 Dec 8;16:271. doi: 10.1186/s13244-025-02156-6 (PMC12686268; doi:10.1186/s13244-025-02156-6)
Supplement: Supplementary file 1 — ELECTRONIC SUPPLEMENTARY MATERIAL [file 13244_2025_2156_MOESM1_ESM.pdf]

Integrating CT-based radiomics and deep learning for invasive prediction of ground-glass nodules in lung adenocarcinoma: a multicohort study

ELECTRONIC SUPPLEMENTARY MATERIAL

S1 CT image acquisition

All CT images are collected using 64-slice spiral CT and above equipment from Siemens, GE, Toshiba, and Philips, while the patient inhales and holds their breath. The scanning range was from the lung apex to the posterior costophrenic angle. The specific scanning parameters were as follows: tube voltage was set at 100 kV or 120 kV, with automatic tube current modulation, a slice thickness of 5 mm, and an image matrix of 512×512. CT images were observed using the lung window (width: 1500HU, level: -600HU) and mediastinal window (width: 350HU, level: 50HU). All CT scan data were reconstructed into thin-section images through post-processing. The data from Zhangjiagang First People's Hospital was reconstructed using the standard algorithm, while the remains were reconstructed using the lung algorithm. The specific scanning and reconstruction parameters are shown in Table S1. Hospital 1: Affiliated Zhongshan Hospital of Dalian University; Hospital 2: Affiliated Xinhua Hospital of Dalian University; Hospital 3: The Second Hospital of Dalian Medical University; Hospital 4: The Fifth People's Hospital of Dalian; Hospital 5: The Affiliated Hospital of Qingdao University; Hospital 6: Zhangjiagang First People's Hospital, respectively.

All CT images were resampled to an isotropic voxel size of 1×1×1 mm^3 to ensure spatial consistency across patients. The intensity values were clipped to the range of -1024HU to 3072 HU to reduce the influence of extreme values and artifacts.

S2 Statistical analysis

This section is a supplement to Section **2.5 Model evaluation** in the main text.

The **ROC** curve (receiver operating characteristic curve) is used to evaluate the performance of a binary classification model. It plots the relationship between the true positive rate (TPR) and the false positive rate (FPR) at different thresholds and quantifies the quality of the model using the area under the curve (AUC).

The **AUC**, intuitively displays the overall ability of the model to distinguish different groups by determining the value, and is the core indicator for evaluating the diagnostic/predictive efficacy of the model.

The **calibration curve** is presented as a line graph of the predicted probability against the actual incidence. Its clinical significance lies in evaluating the absolute accuracy of the model's predicted risk, which is crucial for individualized risk assessment and treatment decisions.

The **DCA** curve (decision curve analysis curve) draws a curve graph of the clinical net benefit under different threshold probabilities. Its core clinical value is to quantify the net clinical benefit and evaluate the practical application value brought by the model under different decision preferences and guide the selection of the optimal decision threshold.

The **NRI** (net reclassification improvement) is used to evaluate the improvement of sample classification accuracy of the new prediction model compared to the old model.

The **IDI** (integrated discrimination improvement) is used to directly compare the differences in the ability of the new and old models to discriminate the probability of outcome events without relying on classification thresholds.

The **Delong test** is used to compare the performance of two classifiers and to determine whether there is a significant difference in the classifier performance based on the difference in AUC values.

Table S1: Systems and scanning parameters of CT image acquisition

Settings	Hospital 1	Hospital 2	Hospital 3	Hospital 4	Hospital 5	Hospital 6
System	Siemens	Siemens	Siemens	Siemens	GE,Siemens, TOSHIBA	GE
Scanning	Helical	Helical	Helical	Helical	Helical	Helical
Tube voltage (kVp)	100	120	120	120	120	120
Tube current (mA)	auto	auto	auto	auto	auto	auto
Pitch	1.2-1.5	0.75-1	0.75-1.5	1.4	0.75-1.5	0.985
Collimation	0.6	0.6	0.6	0.6	0.6	0.625,1.25
Matrix	512 x 512	512 x 512	512 x 512	512 x 512	512 x 512	512 x 512
Pixel pitch (mm)	0.6-1	0.6-1	0.64-0.96	0.75	0.6-1	0.625
Reconstruction algorithm	lung	lung	lung	lung	lung	standard
Slice thickness (mm)	1	1	1	1.5	1-1.25	1.25

S3 Computer configuration and programming environment

The experiments were performed on a workstation equipped with Windows 11 operating system, an Intel i9-13900K processor, 128 GB DDR5 5600 MHz RAM, and an NVIDIA RTX 4090 GPU (24 GB VRAM). GPU acceleration was utilized for all deep learning model training. The models were implemented in Python 3.7.12, using the PyTorch framework (version 1.8.1). Machine learning and statistical analyses were conducted with scikit-learn (version 1.0.2) and statsmodels (version 0.13.2). Image preprocessing and feature extraction were performed using scikit-image (0.19.2), and PyRadiomics (3.0.1). Visualization and plotting were carried out using Matplotlib (3.5.1) and Seaborn (0.11.2).

S4 Radiomics

S4.1 Feature screening

T-test analysis showed that 1515 of the 1834 radiomic features had significant differences between the invasive and non-invasive groups (p<0.05). using the Pearson correlation, the feature set was was reduced from 1515 to 329, effectively removing redundant information (correlation coefficient>0.9). Subsequently, the least absolute shrinkage and selection operator (LASSO) regression algorithm was applied, screening out 53 radiomic features (λ = 0.011, Figure S1) to build the radiomic model. Combined the GridSearch algorithm and five-fold cross-validation, the hyperparameter combination with the best performance was determined for the machine learning models, for example, random forest (RF), extremely randomized trees (ExtraTrees), and light gradient boosting machine (LightGBM). The performance metric for model selection was the mean area under the ROC curve (AUC) across the five folds. The specific training parameters were set as follows: RF (n_estimators=3, max_depth=2, min_samples_split=2); ExtraTrees (n_estimators=3, max_depth=2, min_samples_split=2); LightGBM (n_estimators=3, max_depth=2).

S4.2 Model performance

By comparing the performance of different models on different cohorts, we found that the ExtraTrees-based and LightGBM-based radiomic models both performed well on various aspects but with different advantages, see Table S2 and Figure S2. For training and validation cohorts, the LightGBM model had the highest AUC values (0.964 and 0.917). On test_dl and test_zjg cohorts, the ExtraTrees model performed better (AUC=0.936, 0.914). The LightGBM model had the best AUC performance on the test_qd cohort (AUC=0.856), while all models in this cohort are relatively low. All models performed well on the training cohort, with AUCs ranging from 0.926 to 0.964. However, there’s a drop in performance across all models for the validation cohort, suggesting possible overfitting on the training data. Performance varied across different test cohorts, with some models showing consistent results (e.g., ExtraTrees) while others fluctuated more. To sum up, ExtraTrees appeared to be the most robust model across different datasets, with consistently high AUC and balanced sensitivity and specificity.

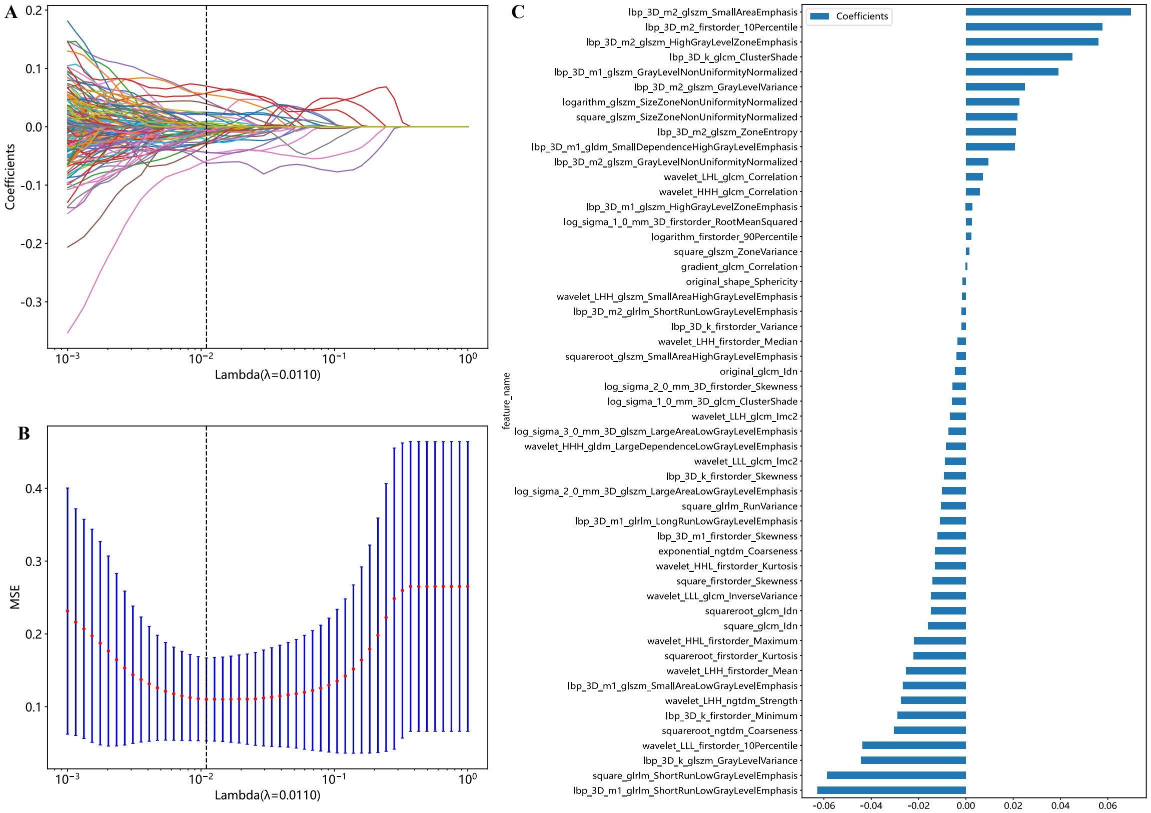

Figure S1: Feature reduction and screening using least absolute shrinkage and selection operator (LASSO) in radiomics. A. Coefficient plot during LASSO cross-validation. B. MSE plot. C. Feature plot of the 53 non-zeros coefficients retained.

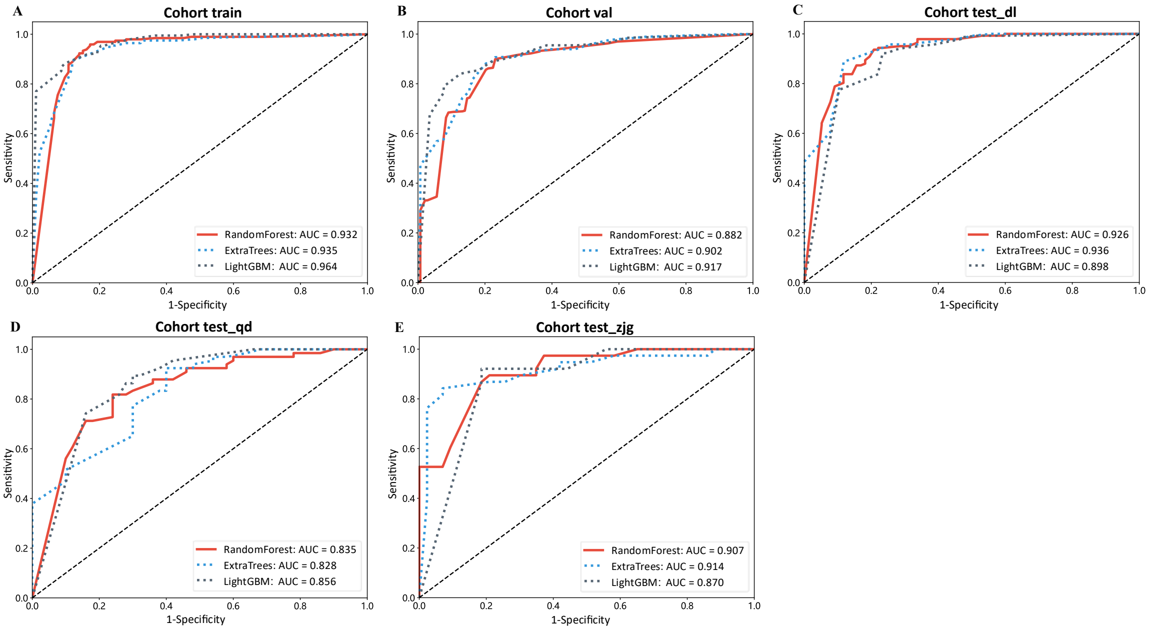

Figure S2: Comparison of receiver operating characteristic (ROC) curves in radiomics using different machine learning classifiers.

Table S2: Performance comparison of radiomics using different machine learning classifiers

model name	ACC	AUC	95% CI	Sensitivity	Specificity	PPV	NPV	Cohort
RandomForest	0.917	0.932	0.896 - 0.968	0.959	0.885	0.922	0.906	train
RandomForest	0.865	0.882	0.849 - 0.916	0.900	0.775	0.916	0.733	val
RandomForest	0.793	0.835	0.760 - 0.910	0.818	0.844	0.818	0.760	test_qd
RandomForest	0.886	0.926	0.886 - 0.965	0.937	0.836	0.893	0.871	test_dl
RandomForest	0.840	0.907	0.846 - 0.968	0.895	0.791	0.791	0.895	test_zjg
ExtraTrees	0.889	0.935	0.903 - 0.966	0.893	0.891	0.941	0.796	train
ExtraTrees	0.860	0.902	0.873 - 0.930	0.882	0.803	0.924	0.708	val
ExtraTrees	0.784	0.828	0.755 - 0.901	0.924	0.600	0.753	0.857	test_qd
ExtraTrees	0.886	0.936	0.903 - 0.969	0.887	0.883	0.933	0.810	test_dl
ExtraTrees	0.889	0.914	0.845 - 0.982	0.842	0.952	0.914	0.870	test_zjg
LightGBM	0.889	0.964	0.944 - 0.983	0.883	0.913	0.951	0.785	train
LightGBM	0.849	0.917	0.891 - 0.943	0.840	0.906	0.949	0.661	val
LightGBM	0.810	0.856	0.787 - 0.925	0.894	0.833	0.797	0.833	test_qd
LightGBM	0.868	0.898	0.853 - 0.944	0.923	0.855	0.879	0.843	test_dl
LightGBM	0.864	0.870	0.797 - 0.943	0.921	1.000	0.814	0.921	test_zjg

[1]AUC: area under the receiver operating characteristic curve; PPV: positive predictive value; NPV: negative predictive value; CI: confidence interval

S5 2.5D deep learning

S5.1 2.5D Model

The flowchart of 2.5D deep learning models is shown in the Figure S3. It is important to note that although we extract features for 7 image slices, we still train the data slice by slice using a 2D deep learning framework, and the 2.5D features (from 7 slices) will be considered in the multiple instance learning (MIL) model (that is why we call it 2.5D). For fairness, the 2.5D model only reports the performance of its benchmark section for patient-level comparison with other models (Table S4), while the MIL-DL model involves the slice-level performance (Figure S4 and Table S3) of all image slices from 2.5D models. To improve generalization capabilities of the models, data augmentation techniques such as random cropping, random brightness adjustment, random rotation, horizontal flipping, and vertical flipping were applied. The models were initialized with ImageNet-pretrained weights, and trained using stochastic gradient descent (SGD) optimizer. The initial learning rate was set to 0.01 and dynamically adjusted using a cosine annealing schedule. The cross-entropy loss was adopted as the objective function. The input images were resized to 224×224 pixels, and a batch size of 64 was used (batch_size=64). The training process was set to a maximum of 30 epochs (epoch=30), with early stopping (patience=6 epochs) to avoid overfitting. Random seeds were fixed to ensure reproducibility.

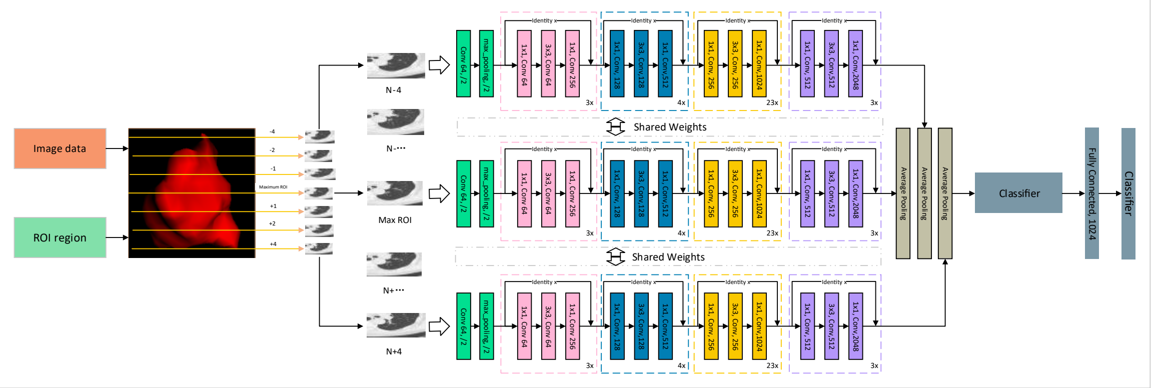

Figure S3: The diagram of 2.5D deep learning model

S5.2 Model performance

In this part, we compared the performance of 2.5D deep learning models including DenseNet201, DenseNet121, ResNet101, ResNet50 and VGG19 on various cohorts. Figure S4 and Table S3 illustrate the performance at the slice level (from all image slices, which will be used for the MIL model), while Table S4 shows the performance at the patient level (only from the benchmark slice, which will be used for comparison with other models to ensure fairness). For the slice level, on the training cohort, DenseNet201 and ResNet50 performed better, with AUC values of 0.907 and 0.893 respectively, while VGG19 showed the lowest AUC value of 0.847; on the validation cohort, DenseNet121, VGG19 and ResNet50 achieved higher AUC values of 0.820, 0.821 and 0.829 respectively, while DenseNet201 ranked last but had an AUC value of 0.816; on the test_qd cohort, DenseNet201 achieved the highest AUC value of 0.816, while VGG19 had an AUC value of 0.814; on the test_dl cohort, ResNet101 had the highest AUC value of 0.894, followed by DenseNet201 and VGG19 with AUC values of 0.882 and 0.875; on the test_zjg cohort, VGG19 had the highest AUC value of 0.843, while ResNet50 had the lowest AUC value of 0.722. Overall, VGG19 demonstrated better performance as it achieved more stable performance on five cohorts with AUC values of 0.847, 0.821, 0.814, 0.875 and 0.843, that is why we choose it for patient-level comparison with other models (Table S4).

Table S3: Performance comparison of 2.5D deep learning models (slice-level)

model name	ACC	AUC	95% CI	Sensitivity	Specificity	PPV	NPV	Cohort
DenseNet121	0.795	0.875	0.8607 - 0.8901	0.855	0.671	0.843	0.690	train
DenseNet121	0.801	0.820	0.8044 - 0.8352	0.877	0.599	0.854	0.647	val
DenseNet121	0.675	0.778	0.7474 - 0.8086	0.588	0.788	0.783	0.596	test_qd
DenseNet121	0.795	0.873	0.8551 - 0.8899	0.851	0.701	0.829	0.734	test_dl
DenseNet121	0.491	0.738	0.6996 - 0.7755	0.989	0.153	0.442	0.952	test_zjg
DenseNet201	0.829	0.907	0.8948 - 0.9195	0.878	0.726	0.869	0.742	train
DenseNet201	0.787	0.816	0.8004 - 0.8315	0.965	0.311	0.789	0.770	val
DenseNet201	0.742	0.816	0.7876 - 0.8439	0.884	0.558	0.722	0.787	test_qd
DenseNet201	0.772	0.882	0.8655 - 0.8989	0.967	0.439	0.746	0.887	test_dl
DenseNet201	0.605	0.783	0.7478 - 0.8175	0.966	0.360	0.506	0.940	test_zjg
ResNet101	0.810	0.883	0.8687 - 0.8971	0.856	0.714	0.861	0.705	train
ResNet101	0.801	0.819	0.8031 - 0.8347	0.946	0.414	0.812	0.744	val
ResNet101	0.726	0.797	0.7672 - 0.8263	0.737	0.712	0.768	0.676	test_qd
ResNet101	0.793	0.894	0.8789 - 0.9092	0.934	0.553	0.780	0.831	test_dl
ResNet101	0.599	0.764	0.7271 - 0.8014	0.914	0.385	0.502	0.868	test_zjg
ResNet50	0.815	0.893	0.8798 - 0.9066	0.865	0.710	0.861	0.717	train
ResNet50	0.801	0.829	0.8148 - 0.8433	0.922	0.476	0.824	0.697	val
ResNet50	0.749	0.804	0.7742 - 0.8332	0.759	0.735	0.788	0.702	test_qd
ResNet50	0.795	0.880	0.8633 - 0.8966	0.906	0.605	0.796	0.791	test_dl
ResNet50	0.581	0.722	0.6824 - 0.7607	0.936	0.339	0.490	0.887	test_zjg
VGG19	0.774	0.847	0.8307 - 0.8637	0.855	0.606	0.818	0.668	train
VGG19	0.797	0.821	0.8071 - 0.8355	0.942	0.410	0.810	0.727	val
VGG19	0.710	0.814	0.7853 - 0.8434	0.657	0.778	0.793	0.636	test_qd
VGG19	0.806	0.875	0.8573 -	0.942	0.573	0.790	0.853	test_dl

VGG19	0.596	0.843	0.8924 0.8096 - 0.8771	0.902	0.388	0.500	0.854	test_zjg

[1]AUC: area under the receiver operating characteristic curve; PPV: positive predictive value; NPV: negative predictive value; CI: confidence interval

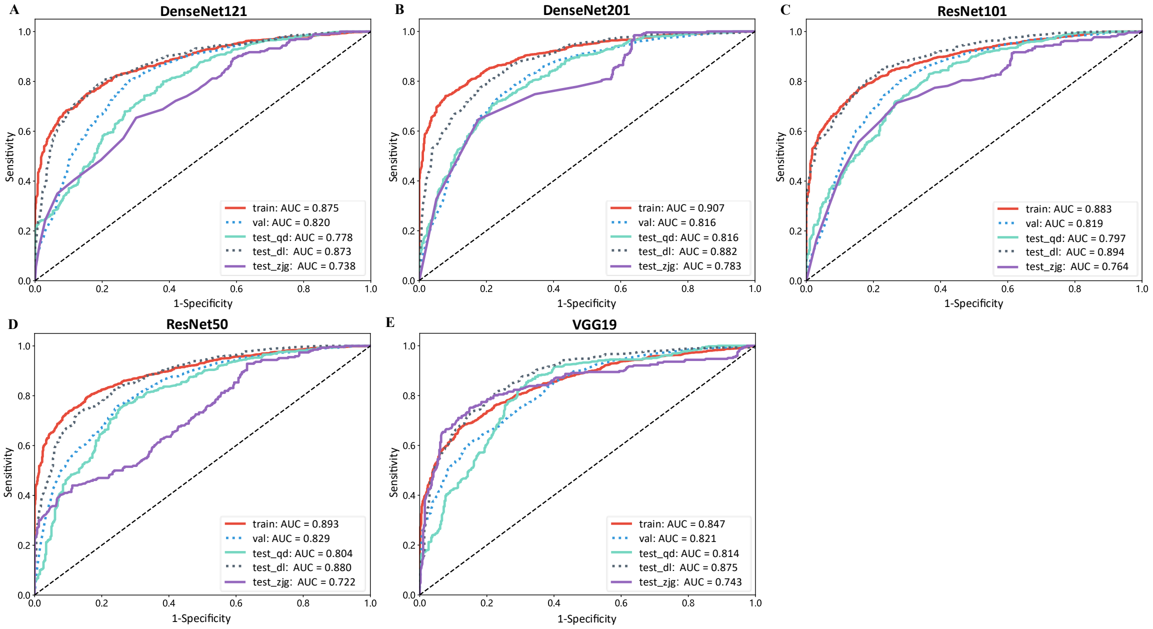

Figure S4: Comparison of receiver operating characteristic (ROC) curves in 2.5D deep learning models (slice-level).

Table S4: Performance comparison of 2.5D deep learning models (patient-level)

model name	ACC	AUC	95% CI	Sensitivity	Specificity	PPV	NPV	Cohort
DenseNet121	0.796	0.900	0.8651 - 0.9351	0.719	0.957	0.972	0.618	train
DenseNet121	0.815	0.860	0.8229 - 0.8975	0.827	0.783	0.914	0.619	val
DenseNet121	0.845	0.920	0.8850 - 0.9545	0.838	0.857	0.915	0.742	test_dl
DenseNet121	0.802	0.859	0.7900 - 0.9273	0.803	0.800	0.841	0.755	test_qd
DenseNet121	0.765	0.816	0.7254 - 0.9074	0.684	0.837	0.788	0.750	test_zjg
DenseNet201	0.841	0.915	0.8826 - 0.9479	0.827	0.871	0.931	0.704	train
DenseNet201	0.828	0.853	0.8131 - 0.8920	0.865	0.727	0.898	0.658	val
DenseNet201	0.849	0.902	0.8609 - 0.9440	0.887	0.779	0.881	0.789	test_dl
DenseNet201	0.802	0.875	0.8146 - 0.9357	0.773	0.840	0.864	0.737	test_qd
DenseNet201	0.815	0.853	0.7699 - 0.9351	0.684	0.930	0.897	0.769	test_zjg
ResNet101	0.754	0.887	0.8499 - 0.9240	0.648	0.978	0.984	0.569	train
ResNet101	0.843	0.847	0.8056 - 0.8889	0.897	0.692	0.891	0.707	val
ResNet101	0.872	0.931	0.8996 - 0.9618	0.894	0.831	0.907	0.810	test_dl
ResNet101	0.802	0.865	0.8001 - 0.9299	0.833	0.760	0.821	0.776	test_qd
ResNet101	0.753	0.798	0.7012 - 0.8949	0.763	0.744	0.725	0.780	test_zjg
ResNet50	0.841	0.914	0.8819 - 0.9459	0.821	0.882	0.936	0.701	train
ResNet50	0.793	0.864	0.8292 - 0.8988	0.799	0.776	0.909	0.581	val
ResNet50	0.845	0.908	0.8691 - 0.9463	0.894	0.753	0.870	0.795	test_dl
ResNet50	0.836	0.877	0.8150 - 0.9398	0.788	0.900	0.912	0.763	test_qd
ResNet50	0.741	0.796	0.7005 - 0.8913	0.632	0.837	0.774	0.720	test_zjg
VGG19	0.723	0.836	0.7896 - 0.8819	0.628	0.925	0.946	0.541	train
VGG19	0.768	0.866	0.8338 - 0.8985	0.754	0.804	0.915	0.540	val
VGG19	0.849	0.930	0.8981 - 0.9624	0.838	0.870	0.922	0.744	test_dl
VGG19	0.845	0.866	0.7964 - 0.9348	0.955	0.700	0.808	0.921	test_qd

VGG19	0.889	0.894	0.8130 - 0.9746	0.789	0.977	0.968	0.840	test_zjg

[1]AUC: area under the receiver operating characteristic curve; PPV: positive predictive value; NPV: negative predictive value; CI: confidence interval

S6 3D deep learning

S6.1 3D Model

The flowchart of 3D deep learning models is shown in the Figure S5. 3D data preprocessing includes several steps as: (1) mask image generation: using the coordinates of the ROI as the center point, a three-dimensional cube mask file was created to enclose the boundaries of the ROI; (2) pixel normalization: the pixel values of each image were sorted, and the intensity was truncated to the range of 0.5 to 99.5 percentile, and then Z-score normalization was performed to reduce the impact of pixel outliers; (3) spatial normalization: fixed-resolution linear interpolation and nearest neighbor interpolation resampling (1mm× 1mm × 1mm) were used for spatial normalization to minimize the impact of voxel-spacing changes; (4) input data generation: based on the coordinate information of the mask file, the cropped 3D ROIs were adjusted to the voxel size of 48 × 48 × 48, and the images were labeled two categories corresponding to the annotated information. To improve model generalization, data augmentation was also applied during training. The models were trained using the adaptive moment estimation optimizer (Adam) with an initial learning rate of 0.001, dynamically adjusted by a cosine annealing schedule. The cross-entropy loss function was adopted as the training objective. A batch size of 4 was used (batch_size=4), and training was performed for up to 96 epochs (epoch=96). Early stopping (patience=6 epochs) was employed to prevent overfitting. The models were randomly initialized, and random seeds were fixed to ensure reproducibility.

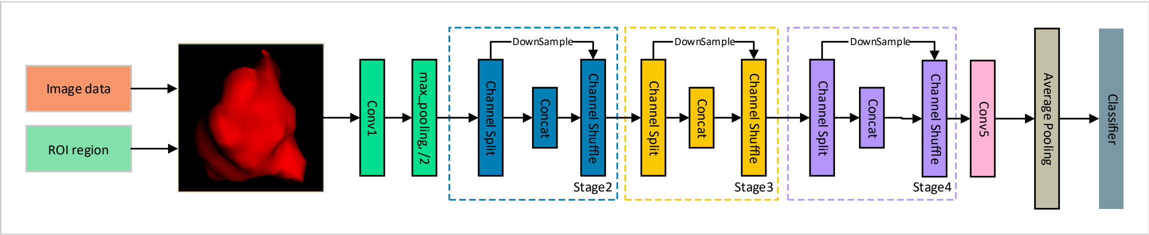

Figure S5: The flowchart of 3D deep learning model

S6.2 Model performance

For 3D deep learning models, we used DenseNet121, ResNet50 and ShuffleNet to compare the performance in predicting the invasiveness of GGN-like pulmonary adenocarcinoma (see Figure S6 and Table S5). On the training and validation cohort, ShuffleNet both had the highest AUC values of 0.992 and 0.802, followed by DenseNet121 with AUC values of 0.934 and 0.787. On the test_qd cohort, DenseNet121 performed the best with an AUC value of 0.792, and ShuffleNet ranked second with an AUC of 0.762. On the test_dl and test_zjg cohorts, ShuffleNet had the highest AUC values of 0.873 and 0.942. Overall, these results indicated that ShuffleNet generally outperformed the other two models across different datasets, especially achieving high sensitivity and specificity. However, DenseNet121 also demonstrated competitive performance, particularly in terms of AUC.

Table S5: Performance comparison of 3D deep learning models

model name	ACC	AUC	95% CI	Sensitivity	specificity	PPV	NPV	Cohort
DenseNet121	0.934	0.969	0.9482-0.9904	0.959	0.882	0.945	0.911	train
DenseNet121	0.792	0.787	0.7404-0.8336	0.872	0.566	0.849	0.614	val
DenseNet121	0.826	0.853	0.7957-0.9105	0.866	0.753	0.866	0.753	test_dl
DenseNet121	0.733	0.792	0.7076-0.8766	0.712	0.760	0.797	0.667	test_qd
DenseNet121	0.877	0.957	0.9179-0.9971	0.789	0.953	0.937	0.837	test_zjg
Resnet50	0.862	0.922	0.8887-0.9555	0.893	0.796	0.902	0.779	train
Resnet50	0.747	0.777	0.7328-0.8220	0.787	0.636	0.858	0.517	val
Resnet50	0.808	0.850	0.7927-0.9080	0.831	0.766	0.868	0.711	test_dl
Resnet50	0.690	0.734	0.6414-0.8265	0.606	0.800	0.800	0.606	test_qd
Resnet50	0.852	0.833	0.7306-0.9346	0.737	0.953	0.933	0.804	test_zjg
ShuffleNet	0.986	0.992	0.9792-1.0000	0.990	0.978	0.990	0.978	train
ShuffleNet	0.795	0.802	0.7597-0.8441	0.880	0.559	0.848	0.625	val
ShuffleNet	0.822	0.873	0.8199-0.9252	0.937	0.610	0.816	0.839	test_dl
ShuffleNet	0.690	0.762	0.6746-0.8490	0.667	0.720	0.759	0.621	test_qd
ShuffleNet	0.852	0.942	0.8828-1.0000	0.711	0.977	0.964	0.792	test_zjg

[1]AUC: area under the receiver operating characteristic curve; PPV: positive predictive value; NPV: negative predictive value; CI: confidence interval

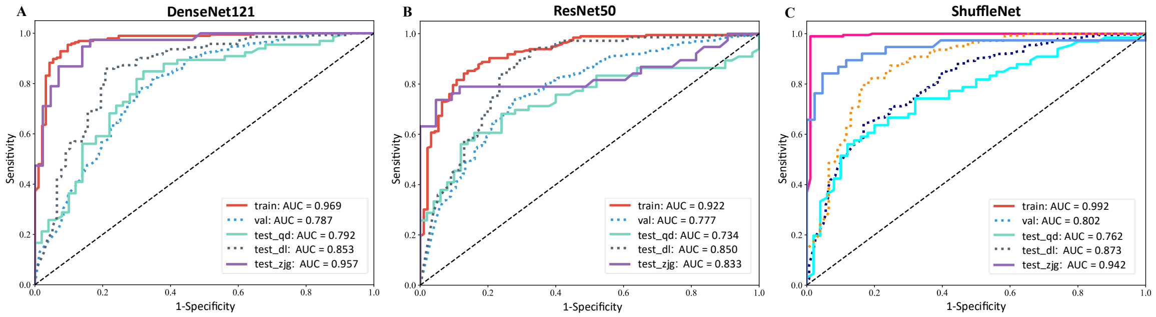

Figure S6: Comparison of receiver operating characteristic (ROC) curves in 3D deep learning models.

S7 MIL-DL model

Here, we established the MIL-DL model by fusing 2.5D and 3D deep learning features using multiple instance learning. Taking the MIL-DL model as an example, for the PLH part, from 2.5D-DL and 3D-DL models, we can obtain the predicted probability and corresponding labels of seven 2.5D slices and 3D VOI in each model, then we can respectively create the probability distribution histograms to represent image features. For the BoW part, predictive results were generated for each sample: 5×7 (5 models, 7 slices) for 2.5D models and 3×1 (3 models) for 3D models. Inspired by the BoW approach, we treated these results analogous to term frequencies in text files and then applied term frequency–inverse document frequency technique to extract the image feature. Finally, the features derived from PLH and BoW were combined to construct a MIL-based feature set, which was used to develop the final MIL-based machine learning models. Combined the GridSearch algorithm and five-fold cross-validation, the hyperparameter combination with the best performance was determined. The performance metric for model selection was the mean area under the ROC curve (AUC) across the five folds. The specific training parameters were set as follows: RF (n_estimators=4, max_depth=2, min samples split=3); ExtraTrees (n_estimators=4, max_depth=2, min samples split=2); LightGBM (n_estimators=5, max_depth=2).

Similar to the flow in radiomics, we used the LASSO regression algorithm to screen out mul-instance features, see Figure S7. We also used the GridSearch algorithm and five-fold cross-validation to learn the optimal hyperparameters for machine learning models. Figure S8 and Table S6 show the predictive results of three machine learning models on various cohorts. On the training cohort, the LightGBM model achieved the best performance with an AUC of 0.989, while all other models also perform very well. On the validation cohort, the RandomForest model had the highest AUC of 0.834, followed by LightGBM model with an AUC of 0.805. Among the different test cohorts, the ExtraTrees model performed the best on test_dl, test_qd, and test_zjg, with AUCs of 0.874, 0.796, and 0.882, respectively. Moreover, the RandomForest model ranked second in AUCs on three test cohorts.

Table S6: Performance comparison of deep learning-based multiple instance learning models

model name	Accuracy	AUC	95% CI	Sensitivity	Specificity	PPV	NPV	Cohort
RandomForest	0.945	0.983	0.969 - 0.997	0.929	0.978	0.989	0.867	train
RandomForest	0.795	0.834	0.792 - 0.876	0.779	0.845	0.931	0.577	val
RandomForest	0.759	0.700	0.595 - 0.805	0.894	0.763	0.737	0.806	test_qd
RandomForest	0.822	0.868	0.815 - 0.921	0.838	0.792	0.881	0.726	test_dl
RandomForest	0.778	0.803	0.710 - 0.896	0.842	0.738	0.727	0.838	test_zjg
ExtraTrees	0.941	0.982	0.971 - 0.993	0.934	0.957	0.979	0.873	train
ExtraTrees	0.756	0.778	0.732 - 0.824	0.747	0.800	0.906	0.526	val
ExtraTrees	0.741	0.796	0.716 - 0.876	0.712	1.000	0.810	0.672	test_qd
ExtraTrees	0.822	0.874	0.825 - 0.923	0.810	0.855	0.906	0.707	test_dl
ExtraTrees	0.877	0.882	0.809 - 0.956	0.921	1.000	0.833	0.923	test_zjg
LightGBM	0.979	0.989	0.979 - 0.999	0.974	0.989	0.995	0.948	train
LightGBM	0.779	0.805	0.768 - 0.842	0.769	0.906	0.916	0.556	val
LightGBM	0.569	0.431	0.352 - 0.510	0.924	0.625	0.575	0.500	test_qd
LightGBM	0.826	0.838	0.784 - 0.891	0.838	0.899	0.888	0.729	test_dl
LightGBM	0.716	0.711	0.602 - 0.819	0.816	0.871	0.660	0.794	test_zjg

[1]AUC: area under the receiver operating characteristic curve; PPV: positive predictive value; NPV: negative predictive value; CI: confidence interval

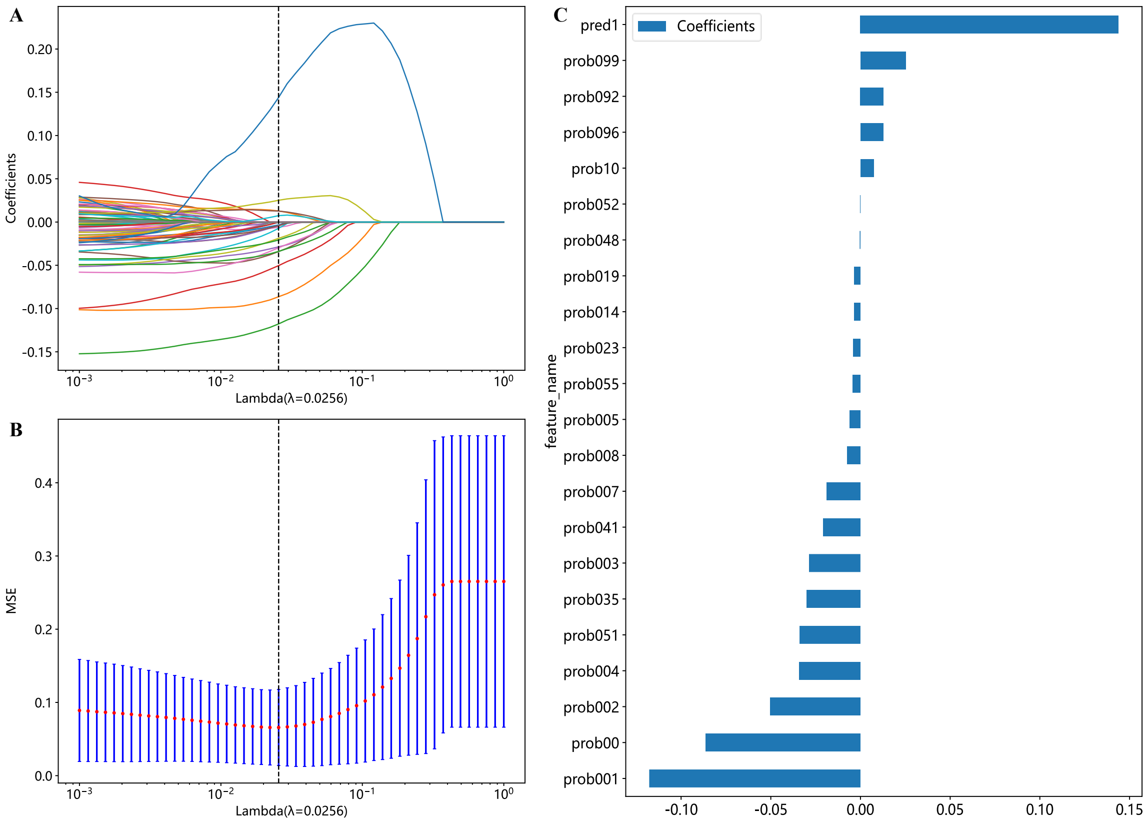

Figure S7: Feature screening in deep learning-based multiple instance learning (MIL-DL) models using least absolute shrinkage and selection operator (LASSO). A. Coefficient plot during LASSO cross-validation. B. MSE cross-validation plot. C. Feature plot of the 22 non-zeros coefficients screened out.

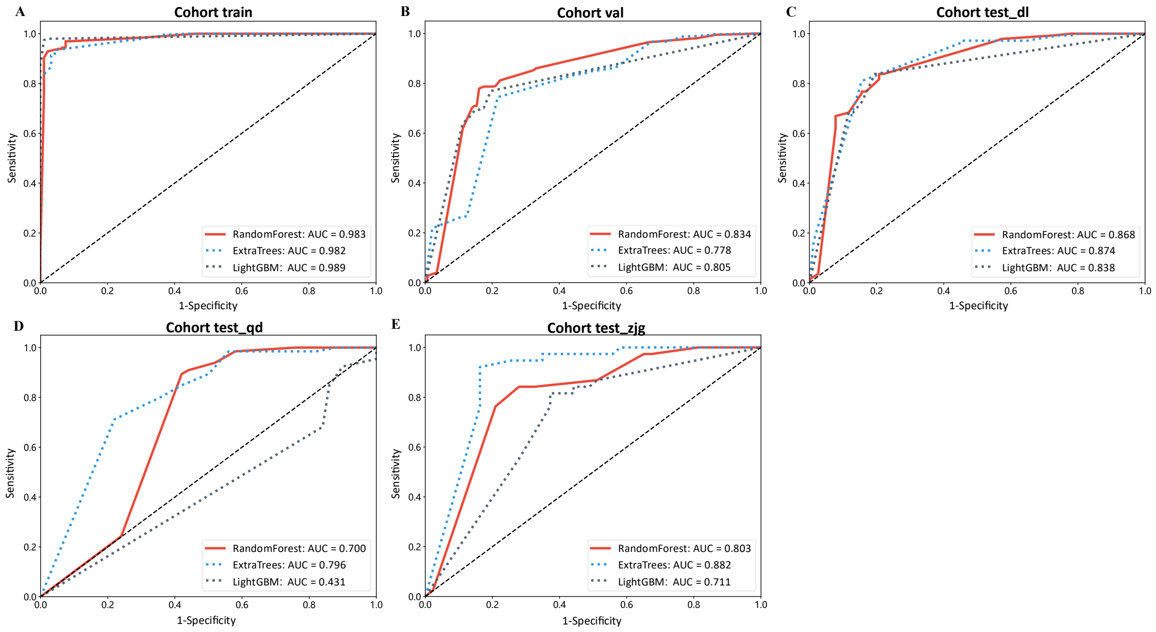

Figure S8: Comparison of receiver operating characteristic (ROC) curves in deep learning-based multiple instance learning (MIL-DL) models

S8 Clinical model extension

For clinical features, based on univariate/multivariate logistic regression analysis, we screened out two independent influencing factors: age and nodule type, with p values less than 0.05. Then, based on two factors, we constructed three clinical prediction models using machine learning classifiers including random forest (RandomForest), extremely randomized trees (ExtraTrees), and light gradient boosting machine (LightGBM). Table S7 and Figure S9 illustrate the predictive results of three machine learning models on various cohorts. RandomForest-based clinical model showed the most stable and high AUC performance on most cohorts (especially the external test sets). ExtraTrees-base model achieved the highest AUC value (0.909) on the test zig cohort. The AUC of LightGBM fluctuated the most among different cohorts, especially on the val and test_qd cohorts, where it performed relatively poorly.

Table S7: Performance comparison of clinical models using different machine learning classifiers

model name	ACC	AUC	95% CI	Sensitivity	Specificity	PPV	NPV	Cohort
RandomForest	0.858	0.889	0.847 - 0.931	0.893	0.820	0.897	0.777	train
RandomForest	0.806	0.866	0.832 - 0.899	0.820	0.775	0.908	0.604	val
RandomForest	0.690	0.764	0.679 - 0.849	0.606	0.833	0.800	0.606	test_qd
RandomForest	0.831	0.897	0.856 - 0.938	0.824	0.855	0.907	0.722	test_dl
RandomForest	0.840	0.907	0.843 - 0.971	0.868	0.814	0.805	0.875	test_zjg
ExtraTrees	0.768	0.863	0.821 - 0.905	0.745	0.817	0.896	0.603	train
ExtraTrees	0.814	0.861	0.827 - 0.894	0.847	0.720	0.894	0.628	val
ExtraTrees	0.741	0.782	0.701 - 0.863	0.773	0.700	0.773	0.700	test_qd
ExtraTrees	0.813	0.883	0.842 - 0.924	0.838	0.766	0.869	0.720	test_dl
ExtraTrees	0.852	0.909	0.849 - 0.969	0.711	0.977	0.964	0.792	test_zjg
LightGBM	0.837	0.885	0.842 - 0.928	0.857	0.871	0.898	0.725	train
LightGBM	0.742	0.849	0.813 - 0.885	0.702	0.924	0.930	0.506	val
LightGBM	0.690	0.683	0.586 - 0.780	0.773	0.744	0.708	0.659	test_qd
LightGBM	0.817	0.879	0.832 - 0.926	0.817	0.863	0.892	0.708	test_dl
LightGBM	0.815	0.835	0.750 - 0.920	0.816	1.000	0.795	0.833	test_zjg

1AUC: area under the receiver operating characteristic curve; PPV: positive predictive value; NPV: negative predictive value; CI: confidence interval

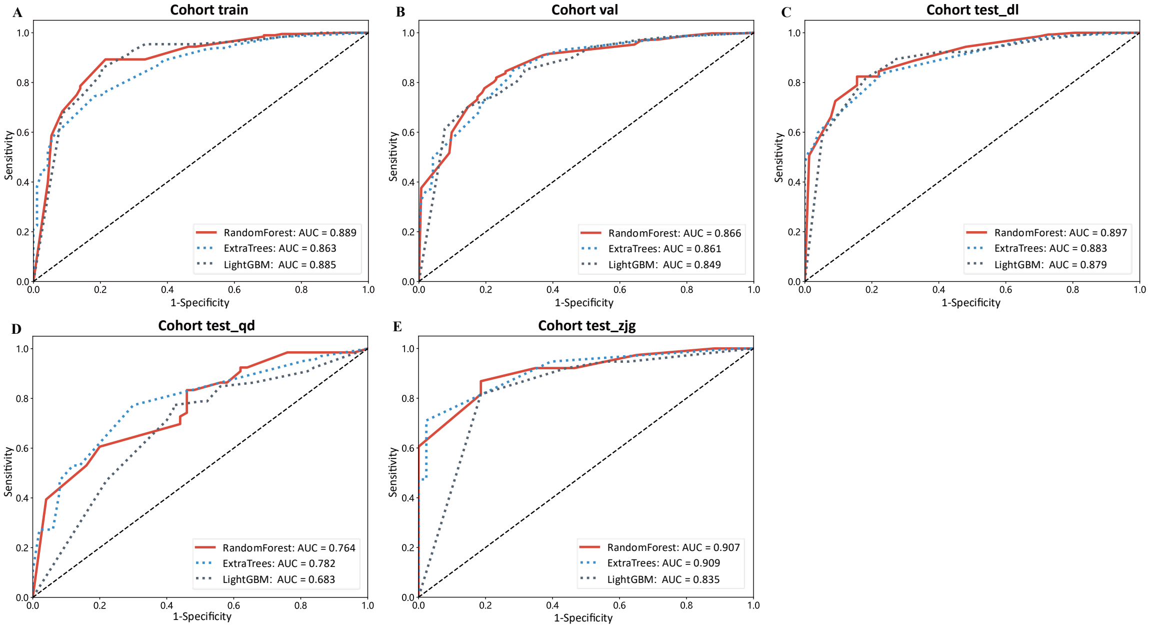

Figure S9: Comparison of receiver operating characteristic (ROC) curves in clinical models

S9 Joint model extension of clinical and MIL-DL-Rad (MIL-DL-Rad+C)

In this part, we also explored the performance of the joint model (termed MIL-DL-Rad+C) by integrating the clinical model and the developed MIL-DL-Rad model in a post-fusion manner using logistic regression model. For the clinical model, we selected RandomForest-based model as it had the most stable and high AUC performance on most cohorts. Table S8 and Figure S10 illustrate the predictive performance comparison of clinical model, MIL-DL-Rad model and the joint MIL-DL-Rad+C model. Incorporating clinical features (i.e., age and nodule type) into MIL-DL-Rad can improve its predictive performance, particularly in the external tes dl (AUC+0.26) and test_zjg (AUC+0.39) cohorts, and the statistical significance was verified by Delong Test (Figure S11). This demonstrates that clinical information can effectively enhance the robustness and discriminative power of the developed model. Moreover, although the AUC increased by 0.35 on the val cohort, the accuracy decreased by 0.42.

Table S8: Performance comparison of clinical, MIL-DL-Rad, and MIL-DL-Rad+C models

model name	Accuracy	AUC	95% CI	Sensitivity	Specificity	PPV	NPV	Cohort
Clinical	0.858	0.889	0.8466 - 0.9308	0.893	0.820	0.897	0.777	train
MIL-DL-Rad	0.886	0.936	0.9020 - 0.9700	0.878	0.913	0.950	0.778	train
MIL-DL-Rad+C	0.886	0.954	0.9287 - 0.9784	0.857	0.946	0.971	0.759	train
Clinical	0.806	0.866	0.8325 - 0.8989	0.820	0.775	0.908	0.604	val
MIL-DL-Rad	0.880	0.881	0.8465 - 0.9163	0.922	0.768	0.915	0.779	val
MIL-DL-Rad+C	0.838	0.916	0.8892 - 0.9427	0.825	0.874	0.948	0.641	val
Clinical	0.831	0.897	0.8558 - 0.9376	0.824	0.855	0.907	0.722	test_dl
MIL-DL-Rad	0.890	0.926	0.8877 - 0.9650	0.944	0.792	0.893	0.884	test_dl
MIL-DL-Rad+C	0.900	0.952	0.9266 - 0.9778	0.944	0.818	0.905	0.887	test_dl
Clinical	0.690	0.764	0.6791 - 0.8491	0.606	0.833	0.800	0.606	test_qd
MIL-DL-Rad	0.810	0.868	0.7987 - 0.9364	0.833	0.780	0.833	0.780	test_qd
MIL-DL-Rad+C	0.828	0.865	0.7955 - 0.9342	0.970	0.640	0.780	0.941	test_qd

Clinical	0.840	0.907	0.8428 - 0.9711	0.868	0.814	0.805	0.875	test_zjg
MIL-DL-Rad	0.864	0.918	0.8591 - 0.9763	0.789	0.930	0.909	0.833	test_zjg
MIL-DL-Rad+C	0.889	0.959	0.9245 - 0.9941	0.789	0.977	0.968	0.840	test_zjg

[1]AUC: area under the receiver operating characteristic curve; PPV: positive predictive value; NPV: negative predictive value; CI: confidence interval

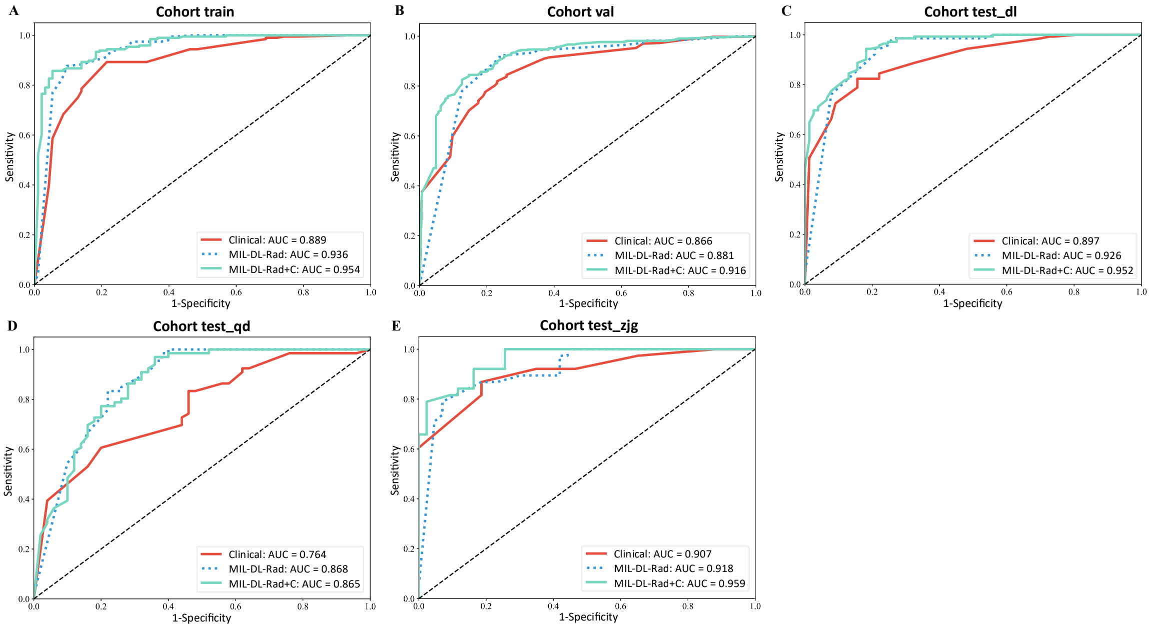

Figure S10: Comparison of receiver operating characteristic (ROC) curves in clinical, MIL-DL-Rad, and MIL-DL-Rad+C models

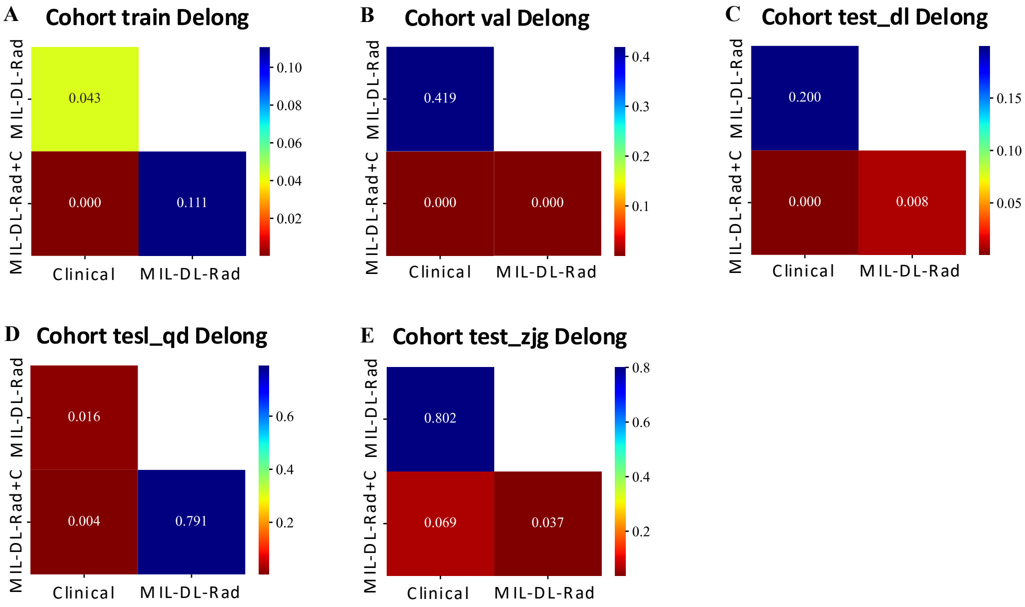

Figure S11: Comparison of Delong test in clinical, MIL-DL-Rad, and MIL-DL-Rad+C model
